# The Role of Mitochondria in Acute Kidney Injury and Chronic Kidney Disease and Its Therapeutic Potential

**DOI:** 10.3390/ijms222011253

**Published:** 2021-10-19

**Authors:** Xiaoqin Zhang, Ewud Agborbesong, Xiaogang Li

**Affiliations:** 1 Department of Internal Medicine, Mayo Clinic, Rochester, MN 55905, USA; Zhang.Xiaoqin@mayo.edu (X.Z.); Agborbesong.Ewud@mayo.edu (E.A.); 2 Department of Nephrology, Tongji Hospital, School of Medicine, Tongji University, Shanghai 200065, China; 3 Department of Biochemistry and Molecular Biology, Mayo Clinic, Rochester, MN 55905, USA; 4 Department of Biochemistry and Molecular Biology, University of Kansas Medical Center, Kansas City, KS 66160, USA

**Keywords:** mitochondria, AKI, CKD, AKI to CKD transition

## Abstract

Mitochondria are heterogeneous and highly dynamic organelles, playing critical roles in adenosine triphosphate (ATP) synthesis, metabolic modulation, reactive oxygen species (ROS) generation, and cell differentiation and death. Mitochondrial dysfunction has been recognized as a contributor in many diseases. The kidney is an organ enriched in mitochondria and with high energy demand in the human body. Recent studies have been focusing on how mitochondrial dysfunction contributes to the pathogenesis of different forms of kidney diseases, including acute kidney injury (AKI) and chronic kidney disease (CKD). AKI has been linked to an increased risk of developing CKD. AKI and CKD have a broad clinical syndrome and a substantial impact on morbidity and mortality, encompassing various etiologies and representing important challenges for global public health. Renal mitochondrial disorders are a common feature of diverse forms of AKI and CKD, which result from defects in mitochondrial structure, dynamics, and biogenesis as well as crosstalk of mitochondria with other organelles. Persistent dysregulation of mitochondrial homeostasis in AKI and CKD affects diverse cellular pathways, leading to an increase in renal microvascular loss, oxidative stress, apoptosis, and eventually renal failure. It is important to understand the cellular and molecular events that govern mitochondria functions and pathophysiology in AKI and CKD, which should facilitate the development of novel therapeutic strategies. This review provides an overview of the molecular insights of the mitochondria and the specific pathogenic mechanisms of mitochondrial dysfunction in the progression of AKI, CKD, and AKI to CKD transition. We also discuss the possible beneficial effects of mitochondrial-targeted therapeutic agents for the treatment of mitochondrial dysfunction-mediated AKI and CKD, which may translate into therapeutic options to ameliorate renal injury and delay the progression of these kidney diseases.

## 1. Introduction

Acute kidney injury (AKI), formerly called acute kidney failure, is defined as kidneys suddenly stopping working properly, ranging from minor loss of kidney function to complete kidney failure and can occur within a few hours or a few days [1]. AKI can also affect other organs such as the brain, heart, and lungs and is associated with significant morbidity [2]. The cause of AKI apart from decreased blood flow, include direct damage to the kidneys and blockage of the urinary tract [3]. Chronic kidney disease (CKD) is defined as the presence of kidney damage persisting over a long period of time, such as three months or more. CKD can cause wastes to build up in your body and can also cause other health problems. The causes of CKD vary globally, and could include diabetes, hypertension, primary glomerulonephritis, chronic tubulointerstitial nephritis, hereditary or cystic diseases, heart diseases, and stroke [4,5]. Even though AKI and CKD were previously thought to be two separate syndromes, they are now recognized to be closely associated or interconnected syndromes, in that AKI is one of the major factors to accelerate the progression of CKD [6], and CKD predisposes or sensitizes patients to AKI [7]. Studies suggest that incomplete or maladaptive repair after AKI leads to tubulointerstitial fibrosis and ultimately to CKD [8,9]. The transition between AKI and CKD represent a global public health challenge [10]. Currently, the treatment of AKI and CKD is predicated on targeting the underlying cause rather than validated specific therapies. Thus, understanding the molecular mechanisms underlying AKI and CKD is crucial for drug development and the generation of novel therapeutic strategies for these kidney diseases.

The kidney is one of the most energy-demanding organs in human and has the second highest mitochondrial content and oxygen consumption after the heart. Mitochondria are a network of plastic organelles that can produce adenosine triphosphate (ATP), thereby supplying the energy source for basal cell functions in the kidneys [11,12]. Mitochondria play an essential role in metabolic modulation, generation of reactive oxygen species, maintenance of intracellular calcium homeostasis, thermogenesis, and regulation of proliferation and intrinsic apoptotic pathways [13,14]. The mitochondria populations can be different in size, mass, metabolic activity, and membrane potential within a cell. Different nephron segments have different mitochondria densities and distributions due to various energy demands. Renal proximal tubular cells require high-energy for reabsorption and secretion against chemical gradients. Proximal tubular cells generate ATP mainly via mitochondrial oxidative phosphorylation, whereas podocytes and endothelial and mesangial cells exhibit more flexibility in their glycolytic capacity to generate energy [15,16]. Accumulating evidence suggests that various acute and chronic injuries may lead to mitochondrial respiratory chain-derived oxidative stress, ultrastructural defects, abnormal activation of the mitochondrial pathway of apoptosis, unstable mitochondrial DNA (mtDNA), and defective clearance of damaged mitochondria. Dysfunction of mitochondria could ultimately increase the risk of tubular interstitial disease, cystic kidney disease, podocytopathy, and nephrotic syndromes [11]. Thus, it is important for us to understand mitochondrial biology and pathophysiology in AKI and CKD. A better understanding of the cellular and molecular events that govern mitochondria functions in kidney diseases should facilitate the development of improved therapeutic strategies. This review provides an overview of the molecular insights of the roles of mitochondria in the progression of AKI, CKD, and the transition from AKI to CKD.

## 2. An Overview of AKI and CKD as Well as AKI to CKD Transition

The term AKI was first used by William MacNider in 1918 in a situation of acute mercury poisoning and has recently been used to replace the term acute renal failure (ARF) [17]. AKI is a broad clinical syndrome that rarely has a sole and distinct pathophysiology, with an incidence of about 2000 per million population. Patients with AKI normally have a mixed etiology, including specific kidney diseases (e.g., acute interstitial nephritis, acute glomerular and vasculitic renal diseases); non-specific conditions (e.g., ischemia, toxic injury); as well as extrarenal pathology (e.g., prerenal azotemia and acute postrenal obstructive nephropathy) [1,18]. The diagnostic approach of AKI at present is based on an acute drop of glomerular filtration rate (GFR) within a short time, which is also reflected with an acute rise in serum creatinine levels and/or a decline in urine output [17,19].

AKI can be classified by three main categories: pre-renal, intrinsic, and post-renal AKI [3,20]. The pre-renal and post-renal AKI are the consequence of extra-renal disease mediated decrease of GFR, whereas ‘intrinsic’ AKI represents true kidney disease. AKI is characterized by renal hemodynamics, renal tubular damage, renal congestion, and inflammation [21]. The pathogenesis of AKI involves in the injury and death of renal tubular cells in the proximal tubule [21,22]. In AKI, the primary site of damage is the plasma membrane, whereas other cellular components, including nucleus, cytoskeleton, endoplasmic reticulum (ER), and mitochondria, are also key targets [22]. A wide array of patient-specific and context-specific factors can increase the risk of AKI, which can be classified as non-modifiable and potentially modifiable factors, including extremes of age, proteinuria, comorbid diseases, anemia, critical illness, sepsis, and fluid overload [23].

CKD is characterized by a gradual loss of kidney function over time and has been defined as eGFR < 60 mL/min/ 1.73 m^2^, irrespective of the presence or absence of kidney damage, and this includes stage 3 CKD (eGFR = 30–59 mL/min/1.73 m^2^), stage 4 CKD (eGFR = 15–29 mL/ min/1.73 m^2^), and stage 5 CKD (eGFR < 15 mL/min/1.73 m^2^ or persons on chronic dialysis), which is also known as end-stage renal disease (ESRD). The definition of ESRD is the final, permanent stage of chronic kidney disease, where kidney function has declined to the point that the kidneys can no longer function on their own [24]. In addition to being a high risk for progression to ESRD, CKD is also an independent risk factor for cardiovascular disease and all-cause mortality. The pathological of CKD features including renal structural and physiological characteristics, as well as the principles of renal tissue injury and repair. Chronic and sustained insults from chronic and progressive nephropathies lead to kidney damage, which is then perpetuated by the process of hyperfiltration and hypertrophy of the remaining nephrons. Initial hyperfiltration activates renin–aldosterone system and causes an increase in arterial filling pressure to the nephrons, resulting in a change in the glomerular architecture and structure as well as in podocytes to further damage the filtration system. Angiotensin II and protein uptake at the tubules causes inflammation and fibrosis of the glomerulus and tubules, leading to a further decrease in renal function. The progression of CKD can be accelerated as a consequence of four mechanisms, including systemic and intraglomerular hypertension, glomerular hypertrophy, intrarenal precipitation of calcium phosphate, and altered prostanoid metabolism [25].

In the last decade, evidence from both clinical and experimental studies suggested a causal link between AKI and CKD, termed as AKI to CKD transition, which is due to incomplete or maladaptive repair after AKI. As AKI has been widely identified as an important risk factor for the occurrence and development of CKD, the potential mechanisms that underlie the progression of AKI and its transition to CKD have also been proposed. They include hypoxia and endothelial dysfunction, nephron loss, alterations of renal resident cell phenotypes and their functions, cell cycle arrest, persistent inflammation, mitochondrial fragmentation, epigenetic modifications, and fibrosis via myofibroblasts recruitment and matrix deposition. In addition, organelle stress signaling caused by ER and mitochondrial stress via induction of tubular cell senescence and subsequent tubular inflammation also contributes to AKI to CKD transition [26,27,28]. The kidney injury molecule-1 (KIM-1) and neutrophil gelatinase-associated lipocalin (NGAL) have been identified as possible biomarkers of AKI to CKD transition [29]. Since prevention of AKI to CKD transition is essential for maintaining kidney function, it is therefore crucial to elucidate the molecular mechanisms underlying this transition, which would facilitate the identification of novel drug targets and the development of novel therapeutic strategies for the treatment.

## 3. An Overview of Mitochondrial Structure, Biogenesis, and Dynamics

### 3.1. Mitochondrial Structure

**Figure 1:**
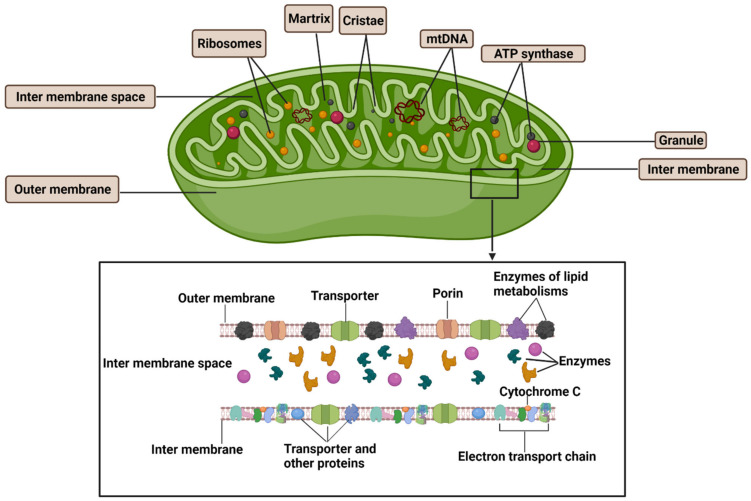
Structure of mitochondria. Mitochondria have an inner and outer membrane with an intermembrane space between them. The outer membrane contains transporters, enzymes for lipid metabolism and proteins (known as porins) that regulate the movement of ions into and out of the mitochondrion. The space within the inner membrane of the mitochondrion is known as the matrix, which contains the DNA, ribosomes, and calcium granules. The inner membrane also contains transporters, a variety of enzymes that regulate the movement of metabolites into and out of the matrix, electron transport chain for energy production via oxidative phosphorylation, and ATP synthase, which generates ATP in the matrix. mtDNA: mitochondria DNA.

Mitochondria are intracellular organelles that contain an inner and outer membrane, with an intermembrane space between them (Figure 1). The outer membrane (OM) of mitochondria connects mitochondria to other cellular organelles, including the endoplasmatic reticulum (ER), the lysosome and the plasma membrane [30]. The OM contains porins, which control the transport of proteins into mitochondria and allows movement of ions in and out of the mitochondria. Enzymes involved in the elongation of fatty acids and the oxidation of adrenaline can also be found on the outer mitochondria membrane. The inner mitochondria membrane (IMM) contains a complex folded structure that increases the total surface area of the IMM and separates two compartments, the intermembrane space, and the matrix. The inner boundary membrane is formed by segments of the IMM, which approximate the OM in close apposition. The cristae structure is a fold in the inner membrane of a mitochondrion, which invaginates into the matrix and harbors the respiratory chain. The tight ridges of the cristae provide enclosed regions that contribute to oxidative phosphorylation to produce ATP, which is an energy source in the cell and transport proteins that regulate the movement of metabolites in and out of the matrix. The space within the inner membrane of the mitochondrion is known as the matrix, where enzymes involved in the Krebs (TCA) and fatty acid cycles, alongside DNA, RNA, ribosomes, and calcium granules, can be found. Mitochondria often undergo transformation in both physiological and pathological conditions. For example, gigantic mitochondria were observed in the cytoplasm of epithelial cells of the proximal tubules in renal biopsies obtained from patients with various renal disorders under electron microscopy [31].

### 3.2. Mitochondrial Dynamics

Mitochondria are highly dynamic organelles that can modulate their morphology to create a tubular network coordinated by fission and fusion events [32]. The balance between these two opposite processes are mainly regulated by large GTPases belonging to the Dynamin family, which regulate mitochondrial number, distribution, and size within the cytoplasm and is referred to as ‘mitochondrial dynamics’, in response to metabolic and signaling cues in the cell environment. Mitochondrial fission is a multi-step process allowing the division of one mitochondrion in two daughter mitochondria mainly mediated by dynamin-related protein 1 (Drp1), a large dynamin-related GTPase. During mitochondrial fission, Drp1 is recruited to the OMM and then GTP hydrolysis enhances this membrane constriction leading to the recruitment of Dynamin 2 to terminate membrane scission. Conversely, mitochondrial fusion is driven by a two-step process caused by several guanosine triphosphate hydrolases (GTPase), including mitofusin 1 (Mfn1) and mitofusin 2 (Mfn2), which contribute to the outer mitochondrial membrane fusion, and optic atrophy 1 (Opa1), which contributes to the inner membrane fusion. Moreover, some membrane lipid components, such as phosphatidic acid and cardiolipin, and several factors that can undergo post-translational modifications, such as Drp1 phosphorylation [33] and SUMOylation/deSUMOylation [34,35], are also involved in the regulation of these processes. The deregulation of these two opposite processes and pathogenic mutations of genes encoding the core fission and fusion machinery components have been linked to AKI and CKD and will be described in detail later.

### 3.3. Mitochondrial Biogenesis

In adaptation to the ever-changing energetic demands triggered by developmental signals and environmental stressors, cells launch the mitochondrial biogenesis process, which takes place mainly in healthy cells. Mitochondrial biogenesis is a complex and multistep process achieved largely through the co-ordination between the mitochondrial and the nuclear genomes that leads to a greater mitochondrial metabolic capacity by increasing synthesis of metabolic enzymes. The mitochondrial DNA (mtDNA) is a double-stranded circular molecule of approximately 16 kb, which encodes 37 genes, including 13 structural subunits of the mitochondrial respiratory chain (electron transport chain complexes I, III, IV, and V) [36]. Mitochondrial biogenesis can be influenced by many environmental stresses like exercise, caloric restriction, and oxidative stress [36]. mtDNA transcription is activated by the family of PPARgamma-coactivator-1 (PGC-1) proteins, including PGC-1α, PGC-1β, and PGC-1. PGC-1α is considered as the master regulator of mitochondrial biogenesis. The induction of mitochondrial biogenesis is associated with the activation of transcription factors and several signaling pathways during tissue injury and repair, including the AMP-activated protein kinase/PGC-1α (AMPK/PGC-1α) pathway used by C1q/tumour necrosis factor-related protein-3 (CTRP3) to promote biogenesis, and the Gβγ (a component of heterotrimeric G proteins)-Akt-eNOS-sGC (soluble guanylatecyclase) pathway stimulated by the β2 adrenergic receptor agonists, such as formoterol [37]. PGC-1α was first identified as a protein interacting with peroxisome proliferator–activated receptor-γ and was highly responsive to cues from environmental factors, which play a critical role in mitochondrial gene expression in a tissue-specific manner [38].

## 4. The Roles of Mitochondrial in AKI, CKD, and AKI to CKD Transition

Mitochondria are very sensitive to changes in environmental factors, which may cause mitochondrial dysfunction [39]. Mitochondrial dysfunction may result in a decrease of ATP generation, an increase of reactive oxygen species level and an induction of apoptosis, all of which contribute to the development and progression of AKI and CKD as well as AKI to CKD transition. Therefore, it is important to understand the roles of mitochondrial biology and pathophysiology in AKI and CKD, which should facilitate novel discoveries for effective therapies of these diseases.

### 4.1. The Roles of Mitochondrial Structure, Dynamics, and Biogenesis in AKI

**Figure 2:**
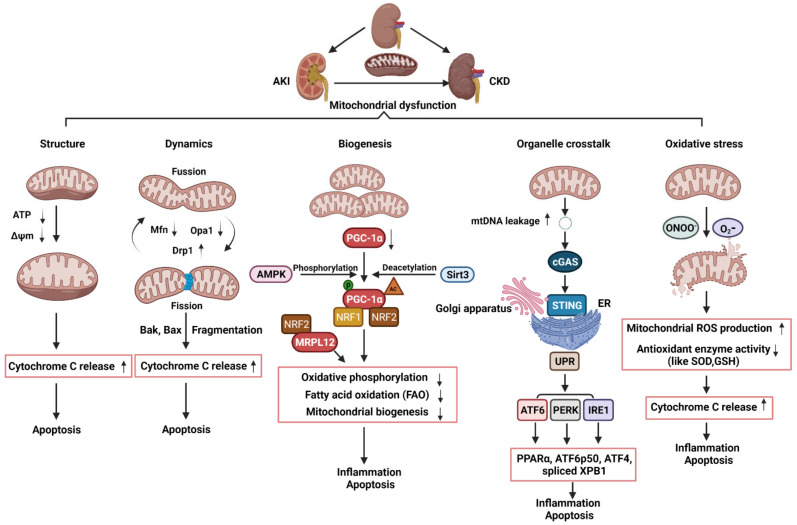
Schematic illustration of pathophysiological processes of mitochondrial dysfunction, including alterations of mitochondrial structure, dynamics, biogenesis, organelle crosstalk, and oxidative stress, in the AKI, CKD, and AKI to CKD transition. AKI: acute kidney injury; CKD: chronic kidney disease; ATP: adenosine triphosphate; ΔΨm: mitochondrial membrane potential; Mfn1: mitofusin 1; Opa1: optic atrophy 1; Drp1: dynamin related protein 1; mtDNA: mitochondria DNA; AMPK: AMP-activated protein kinase; PGC-1α: PPARgamma-coactivator-1α; Sirt3: sirtuin 3; NRF1: nuclear respiratory factor 1; NRF2: nuclear respiratory factor 2; MRPL12: mitochondrial ribosomal protein L12; cGAS; cyclic guanosine monophosphate–adenosine monophosphate (GMP–AMP) synthase; STING: stimulator of interferon genes; ER: endoplasmic reticulum; UPR: unfolded protein response; PPARα: peroxisome proliferator–activated receptor-α; IRE1: inositol-requiring enzyme 1; PERK: PRKR-like ER kinase; ATF6α activating transcription factor 6α; XPB1: the X-box binding protein 1; eIF2α: eukaryotic initiation factor 2α; ATF4: activating transcription factor 4; ONOO-: peroxynitrite; O2-:superoxide; SOD: superoxide dismutase; GSH: glutathione; ROS: reactive oxygen species.

Mitochondria has been recognized as a critical player in AKI with dual roles as the primary source of energy for each cell and as a key regulator of cell death. First, the changes of mitochondria structure have been observed in AKI. Ischemia as a leading cause of AKI diminishes the amounts of mitochondria and induces mitochondria structural changes, typically swelling and showing the disappearance of the inter mitochondrial membrane cristae, due to ATP depletion and membrane potential reduction [40] (Figure 2). In addition, the opening of mitochondrial permeability transition pores (mPTP) due to mitochondrial swelling and dysfunction is a key event that contributes to AKI progression through releasing pro-apoptotic mediators, including cytochrome c, which can induce renal cell apoptosis [41]. mPTP is a nonspecific channel for signal transduction or material transfer between mitochondrial matrix and cytoplasm. In ADR-induced nephropathy rats, treatment with mPTP inhibitor cyclosporine A (CSA) significantly inhibited cytochrome c release and cell apoptosis as well as improved mitochondrial function [42].

Second, the disruption of the balance between fission and fusion events has been associated with AKI progression. The protein Drp1 that could regulate mitochondrial fission was rapidly activated while the proteins, Mfn and Opa1, which could regulate mitochondrial fusion, were decreased following AKI, resulting in mitochondrial fragmentation [43,44] (Figure 2). Cellular stress leads to the oligomerization of Bax and Bak, two proteins of the pro-apoptotic Bcl-2 family, which are susceptible to insert into fragmented mitochondria and consequently outer membrane permeabilization [45]. The permeabilization of mitochondrial outer membrane (MOMP) by pro-apoptotic Bcl-2 family proteins results in the release of apoptogenic factors, such as cytochrome c, which further bind apoptotic peptidase activating factor 1 (Apaf-1) to recruit and activate caspase 9 to trigger the intrinsic apoptotic pathway [46,47,48] (Figure 2). Knockout of Bax or Bak prevented mitochondrial fragmentation along with suppressed cytochrome c release in AKI [49]. Inhibition of mitochondrial fragmentation pharmacologically or by genetically preventing inflammation and cell death shows a significant renoprotective effect in AKI model induced by ischemic reperfusion or nephrotoxic [44,49,50,51].

Third, the dysregulation of mitochondrial biogenesis has also been observed in AKI. In kidneys, PGC-1α is predominantly expressed in proximal tubules and drives mitochondrial biogenesis by its transcriptional co-activators, such as nuclear respiratory factor 1 (NRF1) and nuclear respiratory factor 2 (NRF2). The target genes of NRF1/2 regulate oxidative phosphorylation, fatty acid oxidation (FAO), and the biogenesis of nicotinamide adenine dinucleotide (NAD+), a central metabolic coenzyme/cosubstrate involved in cellular energy, which refer to oxidative metabolism to renal protection [52,53,54,55] (Figure 2). PGC-1α coordinately upregulates the enzymes that synthesize NAD de novo from amino acids, whereas PGC-1α deficiency or AKI attenuates the de novo pathway. The effect of PGC-1α and its association with mitochondrial biogenesis in AKI was supported by the results generated from in Pgc1α^-/-^ mice following ischemia-reperfusion injury [54]. In cisplatin-induced AKI, treatment with AMPK activator 5-aminoimidazole-4-carboxamide-1-β-d-ribofuranoside (AICAR) and the antioxidant agent acetyl-l-carnitine (ALCAR), which activates sirtuin3 (SIRT3) increased PGC-1α activity and improves renal function [56]. Global or tubule-specific PGC-1α knockout mice had normal basal renal function but suffered persistent injury due to prolonged sepsis [57]. In sepsis-associated AKI, endotoxic insults selectively suppress the expression of PGC-1α and then affected PGC-1a mediating the recruitment of NRF1 and NRF2 on genes that regulate oxidative phosphorylation [57,58] (Figure 2). Mitochondrial FAO in proximal tubular cells is a major source of ATP generation. The impairment of mitochondrial FAO has been linked to ATP depletion-induced AKI, and its long-term sequelae leads to CKD [59]. In cisplatin-induced AKI, downregulation of PGC-1α decreased the transcription of FAO genes, including carnitine O-palmitoyltransferase and medium chain–specific acyl-CoA dehydrogenase, leading to decreased mitochondrial FAO (Figure 2). Together, these pieces of evidence indicate that kidney repair or recovery from AKI is associated with mitochondrial structure, dynamics, and biogenesis.

### 4.2. The Roles of Mitochondrial Dynamics and Biogenesis in CKD

Defective mitochondrial dynamics plays an important role in CKD [60]. In experimental models of diabetic kidney disease (DKD) and in kidney biopsy subjects from patients with DKD, tubular cells and podocytes showed an increase in mitochondrial fragmentation [61,62]. In DKD mice, Drp1 was phosphorylated at serine 600 (p-Drp1S600) and mutation of this serine to alanine exhibited improved biochemical and histological features of diabetic nephropathy. Drp1S600 mutation reduced mitochondrial fission and diminished mitochondrial reactive oxygen species (mtROS), further highlighting the stimulus-specific consequences of Drp1 Serine 600 phosphorylation in mitochondrial fission and progression of DKD [63]. Consistent with these findings, pharmacological inhibition, or knockout of Drp1 ameliorated DKD progression as seen with reduced albuminuria, mesangial matrix expansion, and improved podocyte foot process [64,65]. In unilateral ureter obstruction (UUO) mice, Drp1 was phosphorylated at serine 616 (p-Drp1S616) and pharmacological inhibition of mitochondrial fission reduced fibroblasts accumulation, and interstitial fibrosis along with decreased mitochondrial fragmentation and mitochondrial ROS, suggesting that inhibition of the phospho-Drp1S616-mediated mitochondrial fission attenuated fibroblast activation and proliferation in renal fibrosis [66]. Altogether, these findings suggest that mitochondrial fragmentation, owing to a loss of mitochondrial dynamics, plays a critical role in the development of CKD and may serve as a therapeutic target for retarding CKD progression.

Defective mitochondrial biogenesis also plays an important role in CKD. The expression of PGC-1α was decreased not only in experimental CKD models, but also in kidneys from CKD patients [67,68,69,70,71]. Moreover, PGC-1α and PGC-1α-dependent mitochondrial gene expression positively correlated with the glomerular filtration rate and negatively correlated with fibrosis [71]. In DKD, deletion of PGC-1α in podocytes significantly reduced mtDNA, which demonstrates the impact of PGC-1α on mitochondrial biogenesis in podocytes. [72] (Figure 2). Transgenic expression of PGC-1α or activation of PGC1-α in both experimental diabetic nephropathy and cultured podocytes decreased diabetes-induced podocytopenia and glomerular oxidative stress along with prevented mitochondrial dysfunction and cell death [68,73]. PGC-1α was also downregulated in different murine models of renal fibrosis, including Notch transgenic mice and folic acid treatment mice. Tubule-specific overexpression of PGC-1α in these mice result in reducing fibrosis and restoring mitochondrial content [71,74]. In addition to PGC-1α, other additional factors have also been proposed to contribute to altered mitochondrial bioenergetics in CKD, including deletions and mutations of mtDNA and the changes of lipid composition of mitochondrial membranes [74]. The transcription of human mtDNA in vitro requires the single-subunit mitochondrial RNA polymerase (POLRMT) and human mitochondrial transcription factor B2, h-mtTFB2 [75,76]. Mitochondrial ribosomal protein L12 (MRPL12) binds and activates POLRMT to positively control the mitochondrial oxidative phosphorylation and mtDNA copy number [77]. In diabetic kidneys, the expression of MRPL12 was decreased, which was correlated with alterations of mitochondrial function via NRF2 signaling pathway [78] (Figure 2). In addition, a recent study in CKD indicates that reduced activity of mitochondrial matrix dehydrogenases in the skeletal muscle leads to mitochondrial oxidative phosphorylation dysfunction, which correlates with glomerular filtration rate [79]. Further analysis revealed the accumulation of uremic toxins in the muscle that was strongly associated with the degree of mitochondrial impairment. In sum, altered mitochondrial biogenesis has an important effect on the development and progression of CKD.

### 4.3. The Roles of Mitochondrial Dysfunction and Its Crosstalk with ER in the Transition of AKI to CKD

Damaged mitochondria release harmful molecules, such as ROS, DNA, and cardiolipin, which can activate NOD-like receptors (NLR) and elevate the levels of proinflammatory cytokines and chemokines, such as IL-18 and IL-1β, to induce persistent renal injury [80] (Figure 2). The innate immune system has been implicated in both AKI and CKD and persistent inflammation after AKI prevents tissue repair and tubular apoptosis. On one hand, inflammation plays a critical role in the initiation and progression of renal fibrosis, when mitochondrial damage persists long after ischemia to sustain chronic inflammasome activation, leading to persistent endothelial injury, podocyte damage, microvascular rarefaction, and ultimately, progressive glomerular and interstitial fibrosis. On the other hand, upon kidney injury, oxidant stress, abundant cytokines, or hypoxia, deteriorate the mitochondrial membrane potential by excreting ROS and releasing pro-apoptotic factors, such as cytochrome c and apoptosis-inducing factor (AIF), which promote caspase dependent and independent apoptosis. In this perspective, persistent mitochondrial dysfunction result in persistent tubular damage, which may affect renal recovery from AKI and further progression to CKD. In a study with AKI to CKD transition experimental model, the investigators performed a long (nine months) follow-up, exploring the role of mitochondria in rats [80]. They confirmed that AKI is not merely an acute phenomenon but results in long-lasting morphologic and functional consequences. AKI induced peritubular and glomerular capillary loss, podocyte damage, and increased profibrotic and proinflammatory cytokines from one to nine months, leading to progressive glomerular and interstitial fibrosis. Transmission electron microscopy revealed major alterations of mitochondria including loss of cristae and matrix density in endothelial cells, podocytes, and tubular cells up to nine months after the injury. Similar data was also presented in the Lan et al. study [40], which showed that persistence in mitochondrial morphologic alterations and significant reductions in mitochondrial number and metabolic dysfunctions at 14 days after IRI plays a key role in the development of renal tubular atrophy and the transition to CKD after AKI. Studies have also focused on the role and mechanisms of impaired protein kinase B (PKB/AKT1) signaling, which works together with mitochondrial proteins, in the regulation of ATP production and oxidative phosphorylation in renal tubular epithelial cells. Mitochondrial AKT1 inhibition led to activation of caspases and tubular cell death, and renal fibrosis after ischemia-reperfusion injury [81]. Altogether, these studies suggest long-term mitochondrial damage may affect the pathophysiology and recovery from AKI and result in the gradual progression to CKD.

In addition, studies show that mitochondrial dysfunction disrupts the crosstalk between mitochondria and ER, leading to tubular inflammation and fibrosis as well as the AKI to CKD transition. ER is a major organelle that controls protein synthesis, folding, and degradation via the unfolded protein response (UPR) pathway. The UPR promotes cellular survival by restoring ER and mitochondrial homeostasis through distinct signaling networks, but if unsuccessful, the UPR induces cell death [82]. UPR pathways is regulated by three distinctive transmembrane sensors: activating transcription factor 6 (ATF6), PRKR-like ER kinase (PERK), and inositol-requiring enzyme 1 (IRE1), which can be activated under ER stress [83] (Figure 2). Various types of kidney damage are associated with dysfunction of the ER and the activation of the UPR. In cisplatin-induced AKI model, cisplatin-induced mitochondrial damage and mtDNA leakage into the cytosol in renal tubular cells, and damaged mtDNA subsequently increased the activation of cyclic guanosine monophosphate–adenosine monophosphate (GMP–AMP) synthase (cGAS)-stimulator of interferon genes (STING) pathway, resulting in the activation of UPR response and then renal inflammation and AKI progression [84]. The activation of ATF6α caused by pathogenic conditions significantly reduces mitochondrial fatty acid β-oxidation activity through suppressing the expression of peroxisome proliferator–activated receptor-α (PPARα) and thereby induced tubular inflammation and fibrosis after acute kidney injury induced by lipotoxicity [70]. Furthermore, activated ATF6 can be translocated to the Golgi apparatus for cleavage to form an active fragment (ATF6 p50). The activation of IRE1 and PERK induces the splicing of the X-box binding protein 1 (XBP1) mRNA and phosphorylates eIF2α, which promotes the translation of activating transcription factor 4 (ATF4) and suppresses the translation of other mRNAs to reduce unfolded proteins, respectively. ATF6 p50, spliced XBP1, and ATF4 could induce the transcription of various UPR target genes that regulate inflammation and apoptosis (Figure 2) [85]. These findings suggest that alterations in ER–mitochondria crosstalk may contribute to the progression of AKI to CKD transition.

## 5. Mitochondrial Oxidative Stress in AKI and CKD

Oxidative stress is considered a common feature of AKI and CKD. There is now an increasing body of evidence to suggest that the generation of reactive oxidative species (ROS) is significantly increased not only in experimental injured kidneys, but also in patients with failing kidneys. The generation of mitochondrial ROS mainly takes place at the electron transport chain located on the IMM during the process of oxidative phosphorylation. Leakage of electrons at electron transport chain complex I (NADH dehydrogenase (ubiquinone)) and complex III (ubiquinol-cytochrome c reductase) leads to partial reduction of oxygen to form superoxide dismutase (O_2_^−^), which is the major ROS production in mitochondria [86]. O_2_^−^ can undergo radical reaction with nitric oxide (NO) to form peroxy nitrite (ONOO^−^) within mitochondrial. To maintain a balanced amount of ROS in the cell, the superoxide dismutase (SOD) family catalyzes the dismutation of O_2_- to hydrogen peroxide (H_2_O_2_) [86]. The H_2_O_2_ is degraded following the intervention of specific enzyme glutathione (GSH). Mitochondrial ROS can induce mPTP, consequently rendering the IMM proteins, such as cytochrome c, to the cytosol to eventually trigger cell inflammation and apoptosis (Figure 2). The main etiologies of AKI are ischemia and hypoxia. Hypoperfusion caused by decreased blood flow results in limitation of the cellular nutrient and oxygen uptake, leading to acute tubular necrosis. Ischemia and reperfusion represent major triggers of ROS, with mitochondria being the primary source of ROS, in renal function and tissue integrity. In sepsis-induced AKI, extensive immune response results in the upregulation of inducible NO synthase and leads to production of excessive NO, which is responsible for endothelial injury, localized hypoxia, and the formation of ROS (Figure 2). Other etiologies of AKI, such as nephrotoxic AKI induced by cisplatin, were also associated with ROS-dependent renal cell death [87,88]. The major site of cisplatin-induced nephrotoxicity is in the proximal tubule cells. The accumulation of cisplatin in the kidney causes mitochondrial structural damage, decreases the levels of antioxidants, such as GSH, and decreases the activity SOD [89] (Figure 2). Moreover, mitochondrial dysfunction inhibits the activity of complexes I to IV of the respiratory chain, resulting in ROS formation and the reduction of ATP generation [89]. Consistent with these findings, studies suggest that the administration of SS-31 peptide (D-Arg-Dmt-Lys-Phe-NH2), a mitochondria peptide, can alleviate key features of AKI, which are related to anti-mitochondrial ROS [88,90].

Mitochondrial oxidative stress in CKD kidneys are also higher compared to healthy kidneys. In a cohort study, Oberg et al. found that 60 patients with stage 3–5 CKD had higher oxidative stress than the healthy subject cohort by comparing the oxidative stress markers (plasma protein carbonyl group content, plasma free F2-isoprostane content, plasma protein reduced thiol content). Moreover, an increase in oxidative stress was noted in diabetic and hypercholesterolemic patients [91]. The increasing oxidative stress in CKD has been associated with dysfunctional mitochondria [13,60,92]. By comparing CKD patients with healthy people, it has been found that CKD/HD patients may have an impaired mitochondrial respiratory system and this condition may be the consequence and the cause of an enhanced oxidative stress by using a high-throughput genomic approach based on a whole transcriptomic analysis associated with classical molecular methodologies [93]. In addition, an experimental model of diabetes, which express a redox-sensitive Green Fluorescent Protein biosensor (roGFP), exhibited a marked increase in mitochondrial reactive oxygen species in the kidneys [92]. Treatment with mitochondria-targeted antioxidants like mitoTEMPO can alleviate podocyte injury and loss in diabetic nephropathy [94]. Furthermore, oxidative status in CKD may adversely affect AKI. For example, in an orthopedic trauma-induced model, obese rats with higher oxidative stress developed more severe AKI [95].

## 6. Mitochondrial Targeting for AKI and CKD Therapy

**Figure 3:**
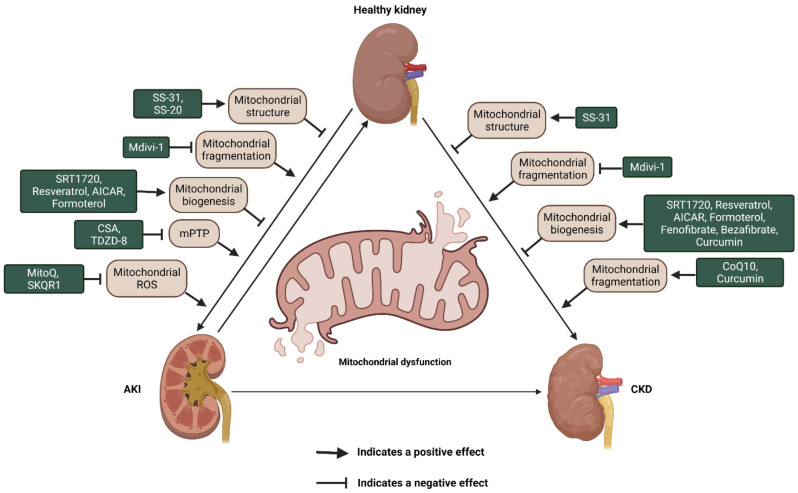
Schematic illustration of the potential compounds that target the dysfunction of mitochondrial structure, fragmentation and biogenesis as well as mPTP opening and mitochondrial antioxidant capacity in AKI and CKD. AKI: acute kidney injury; CKD: chronic kidney disease; ROS: reactive oxygen species. mPTP: mitochondrial permeability transition pore; CSA: Cyclosporine-A; TDZD-8: 4-benzyl-2-methyl-1,2,4-thiadiazolidine-3,5-dione; CoQ10: Coenzyme Q10 MitoQ: mitochondrial coenzyme Q; SkQR1: 10-(6′-plastoquinonyl) decylrhodamine 19.

Given the abundant evidence for a critical role of mitochondrial dysfunction in various acute and chronic kidney injuries, mitochondria, thus, have been recognized as a promising target to improve treatment of patients with kidney diseases. Currently, numerous novel mitochondria-targeted compounds are being exploited in AKI and CKD. Based on the roles and mechanisms, these agents have been classified as compounds for cardiolipin protection, inhibitors of mitochondrial fragmentation, compounds for promoting mitochondrial biogenesis, and inhibitors of mPTP and mitochondria oxidants (Figure 3 and Table 1).

### 6.1. Cardiolipin Protection Agents

Cardiolipin (CL), one of the major phospholipids localized and synthesized in the inner mitochondrial membrane was first isolated from beef heart in the early 1940s [125]. CL interacts with and is required for optimal activity of several IMM proteins, including the enzyme complexes of the electron transport chain and ATP production [126]. Its deficiency results in mitochondrial dysfunction, including the changes of mitochondrial membrane morphology, stability and dynamics, mitochondrial biogenesis, and protein import, as well as a decrease in respiratory performance and an increase in ROS generation [126,127]. Ischemia could induce mitochondria degenerative changes with matrix swelling and loss of the IMM cristae in all renal cells due to ATP depletion and dysregulation of osmotic influx of water [128]. Given that mitochondrial structure and function are closely linked, a promising target to preserve mitochondrial structure might contribute to maintain normal mitochondrial function and improve mitochondrial function following ischemia. The development of compounds to protect cardiolipin and to optimize efficiency of the electron transport chain and thereby restore cellular bioenergetics has been an innovative discovery. Szeto-Schiller (SS) peptides are among novel mitoprotective drugs. SS-31 (also known as elamipretide) as a synthetic tetrapeptide (d-Arg-2′6′-dimethylTyr-Lys-Phe-NH2) can selectively bind to CL through electrostatic and hydrophobic interactions on the inner mitochondrial membrane to protect cristae curvature, stabilize mitochondrial structure, facilitate the transport of electrons, and minimize ROS production, which has been reported to attenuates cardiac damage in experimental models of heart failure [129,130]. SS-31 has been tested in experimental AKI and CKD. In ischemic reperfusion-induced AKI model, administration of SS-31 before the injury protected endothelial and epithelial mitochondria, preserved peritubular and glomerular capillaries, and prevented inflammation [131]. In another study, treatment with SS-31 one month after ischemia and maintaining for six weeks showed the protection of mitochondrial integrity, restoration of peritubular and glomerular capillaries, preservation of podocyte architecture, and suppression of inflammation and the progression of glomerulosclerosis and tubular interstitial fibrosis [80], suggesting that SS-31 showed a renoprotective by restoring mitochondrial structure and function, inhibiting proinflammatory and profibrotic response in AKI to CKD transition. In CKD models, treatment with SS-31 overcame lipotoxicity, glomerulosclerosis, and tubular lesions and injury in kidneys induced by a high-fat diet and urinary tract obstruction (UUO) [96,97,98]. Treatment with SS-31 improves renal function either by accelerating the recovery of ATP or scavenging ROS and suppressing mitochondrial permeability transition. In addition to SS-31, another SS family drug, SS-20, has also been tested in AKI and CKD. Treatment with SS-20 could increase the efficiency of the electron transport chain and improve the coupling of oxidative phosphorylation, which are also considered to be mechanisms that reduce IR injury. SS-20 could reduce mitochondrial matrix swelling and preserved cristae membranes, which enhanced mitochondrial ATP synthesis under ischemic conditions. Treatment with SS-20 could also significantly reduce renal interstitial fibrosis after ischemia [99].

### 6.2. Modulating Mitochondrial Dynamics

Mitochondrial fission is governed by Drp1, the inhibition of which, attenuates renal tubular injury and subsequent progression of AKI induced by ischemia reperfusion and cisplatin [44,100]. Proximal tubule–specific deletion of Drp1 not only prevented the renal ischemia-reperfusion–induced inflammation and programmed cell death, but also attenuated progressive kidney injury and fibrosis [51]. Therefore, targeting Drp1 might be beneficial in the treatment of diseases considering mitochondrial fission alteration. Mitochondrial division inhibitor-1 (Mdivi-1), a pharmacological inhibitor of Drp1 via blocking its assembly and GTPase domain activity [132], blocked mitochondrial fragmentation. During cell injury, the balance between mitochondrial fusion and fission shifts to mitochondrial fission, which results in mitochondrial fragmentation [133]. Mdivi-1 treatment has been tested in different AKI models. In rhabdomyolysis (RM)-induced AKI, Mdivi-1 treatment ameliorates renal function by maintaining the mitochondrial function and reducing the apoptosis of tubular cells [100]. In sepsis-induced AKI, Mdivi-1 treatment had renoprotective effect due to protection of mitochondrial function characterized by increasing ATP production and decreasing mitochondrial fragmentation, and the reduction of NOD-like receptor pyrin domain-3 (NLRP3) inflammasome-mediated pyroptosis of renal tubular epithelial cells [134]. Mdivi-1 also has been tested in CKD models; however, Mdivi-1 treatment seems to exhibit divergent functions in different studies. In Ksp-Cre;Pkd1^flox/-^ mice, administration of Mdivi-1 significantly reduced kidney/body weight, cyst formation, and improved renal function by interfering with Drp1 and rescuing mitochondrial fragmentation [101]. In UUO model, treatment with Mdivi-1 decreased mitochondrial PTEN-induced putative kinase 1 (PINK1), parkin RBR E3 ubiquitin protein ligase (PARK2), and LC3II levels; increased mtROS production; and worsened renal fibrosis following UUO [102], which suggested that Drp1-regulated PARK2-dependent mitophagy plays a protective role in kidney following injury. Therefore, it is hard to evaluate whether Midvi-1 treatment is a benefit or not and more evidence for the application of Mdivi-1 is needed in CKD.

### 6.3. Altering Mitochondrial Biogenesis

Mitochondria biogenesis refers to a process of generating new mitochondrial mass and replicating mtDNA, in which, activation is necessary for the increased metabolism and energy demands during the recovery from acute organ injury. The AMPK/SIRT/PGC-1α axis plays crucial roles in mitochondrial biogenesis. As mentioned above, PGC-1α have been identified as a critical regulator that coordinately regulates mitochondrial biogenesis, reduces oxidative stress, and anti-inflammatory [54]. The expression and activity of PGC-1α can be regulated by Sirtuin1 (SIRT1), a NAD-dependent deacetylase. In IRI-AKI, treatment with SIRT1 activator, SRT1720 restores the expression of PGC-1α in kidneys, leading to enhanced mitochondria biogenesis characterized by the increase of mitochondrial mass and ATP levels, and improved renal function [103]. Other activators of mitochondria biogenesis include resveratrol, AICAR, and formoterol. Treatment with resveratrol [105], a natural plant phytoalexin, protects mice against aldosterone-induced podocyte injury by upregulating PGC-1α and restores mitochondrial respiratory capacity and decreases the production of mitochondria ROS and lipid peroxidation in AKI [68,135]. Pretreatment with AICAR, an AMPK activator, attenuated I/R injury-induced nitrosative stress and monocyte/macrophage infiltration, and ameliorated the development of acute tubular necrosis [108]. Formoterol is a potent agonist of β2-adrenoreceptor, which can induce mitochondrial biogenesis by increasing mtDNA copy numbers, oxygen consumption rate, and PGC-1α expression in both the renal proximal tubular cells and cardiomyocytes [110]. Treatment with formoterol rescued injury and tubular necrosis, decreased nitrosative stress, and ameliorated renal function in IRI-AKI [111]. All these drugs have also been tested in CKD models. For example, either SRT1720 or AICAR treatment exerted protective effects against tubulointerstitial fibrosis by decreasing oxidative stress [104,109]. In diabetic nephropathy, treatment with resveratrol prevents lipotoxicity-related apoptosis and oxidative stress [105,106,107]. Formoterol treatment was also shown to be renoprotective in diabetic nephropathy, which reduced proteinuria and kidney profibrotic proteins by restoring mitochondrial fusion/fission protein level [112]. In addition, another group of drugs known as “fibrates”, including bezafibrate and fenofibrate, which could increase mitochondrial fatty acid oxidation and mitochondrial biogenesis, have also been shown to have renoprotective effects in diabetic experimental models [113]. Thus, mitochondria biogenesis activators can be promising therapeutic targets.

### 6.4. Altering Mitochondrial Biogenesis

Mitochondria biogenesis refers to a process of generating new mitochondrial mass and replicating mtDNA, of which, activation is necessary for the increased metabolism and energy demands during the recovery from acute organ injury. The AMPK/SIRT/PGC-1α axis plays crucial roles in mitochondrial biogenesis. As mentioned above, PGC-1α have been identified as a critical regulator that coordinately regulates mitochondrial biogenesis, reduces oxidative stress, and is anti-inflammatory [54]. The expression and activity of PGC-1α can be regulated by Sirtuin1 (SIRT1), a NAD-dependent deacetylase. In IRI-AKI, treatment with SIRT1 activator SRT1720 restores the expression of PGC-1α in kidneys, leading to enhanced mitochondria biogenesis characterized by the increase of mitochondrial mass and ATP levels, and improved renal function [103]. Other activators of mitochondria biogenesis include resveratrol, AICAR, and formoterol. Treatment with resveratrol [105], a natural plant phytoalexin, protects mice against aldosterone-induced podocyte injury by upregulating PGC-1α and restores mitochondrial respiratory capacity and decreases the production of mitochondria ROS and lipid peroxidation in AKI [68,135]. Pretreatment with AICAR, an AMPK activator, attenuated I/R injury-induced nitrosative stress and monocyte/macrophage infiltration, and ameliorated the development of acute tubular necrosis [108]. Formoterol is a potent agonist of β2-adrenoreceptor, which can induce mitochondrial biogenesis by increasing mtDNA copy numbers, oxygen consumption rate, and PGC-1α expression in both the renal proximal tubular cells and cardiomyocytes [110]. Treatment with formoterol rescued injury and tubular necrosis, decreased nitrosative stress, and ameliorated renal function in IRI-AKI [111]. All these drugs have also been tested in CKD models. For example, either SRT1720 or AICAR treatment exerted protective effects against tubulointerstitial fibrosis by decreasing oxidative stress [104,109]. In diabetic nephropathy, treatment with resveratrol prevents lipotoxicity-related apoptosis and oxidative stress [105,106,107]. Formoterol treatment was also shown to be renoprotective in diabetic nephropathy, which reduced proteinuria and kidney profibrotic proteins by restoring mitochondrial fusion/fission protein level [112]. In addition, another group of drugs known as “fibrates”, including bezafibrate and fenofibrate, which could increase mitochondrial fatty acid oxidation and mitochondrial biogenesis, have also been shown to have renoprotective effects in diabetic experimental models [113]. Thus, mitochondria biogenesis activators can be promising therapeutic targets.

### 6.5. mPTP Inhibitors

Opening of high conductance mPTP initiates onset of the mitochondrial permeability transition (MPT) and leads to cellular necrosis and apoptosis after oxidative stress, Ca^2+^ toxicity, and ischemia/reperfusion [136]. mPTP putatively consists of the voltage-dependent anion channel, the adenine nucleotide translocator (ANT), and cyclophilin D. The inhibitors of mPTP have been shown to ameliorate renal IRI and drug-induced AKI [42,114]. Cyclosporine-A (CSA), a potent inhibitor of the mPTP, blocking the interaction of cyclophilin D with the ANT, results in blocking the conformational change of ANT [137], which is currently under clinical trial for its effect on reperfusion injury on acute myocardial infarction (NCT01595958) and severe traumatic brain injury (NCT01825044). Low dose of CSA suppressed mPTP opening and mitochondria swelling, reduced podocyte damage [42], attenuated the oxidative stress, and worked against subsequent ischemia/reperfusion injury [114]. However, high-dose CSA treatment results in nephrotoxicity and apoptosis by shifting mitochondrial dynamics toward fission [138] and specific metabolic changes, including the decrease of the activity of the mitochondrial Krebs cycle, oxidative phosphorylation [139], electron transferring, and the shutting down of oxidative aerobic pathway [140]. These studies suggested a dosage limitation usage of CSA in patients with renal disease. Notably, in db/db mice, treatment with cyclophilin D inhibitor alisporivir did not improve renal function nor pathology in kidneys indicating that cyclophilin D has a complex role in DKD and direct targeting of this component of the mPTP will likely not improve renal outcomes [141]. Thus, the value of mPTP inhibitors application in CKD needs further investigation to be evaluated. Other agents like 4-benzyl-2-methyl-1,2,4-thiadiazolidine-3,5-dione (TDZD-8), a selective inhibitor of glycogen synthase kinase 3β (GSK-3β), which is a ubiquitous serine–threonine protein kinase that phosphorylates cyclophilin D and promotes mPTP opening, also prevent acute kidney dysfunction in AKI [115,116,117]. Moreover, TDZD-8 could suppress renal fibrosis developed as a result of acute kidney injury, which represents a therapeutic approach for AKI to CKD transition [117].

### 6.6. Mitochondrial-Targeted Antioxidants

The mitochondrion is a main source of intracellular ROS, in that ~90% of ROS are generated in mitochondria [16]. The burst of mtROS has been shown to disturb multiple pathways involved in calcium homeostasis, mitochondrial permeability, cytochrome C release, activation of proinflammatory signals, such as Toll-like receptors (TLRs) and the NLRP3 inflammasome, and to directly induce renal cell death [11,142,143,144]. Considering the increased mtROS generation in AKI and CKD, drugs that specifically target mtROS may confer greater protection against renal injury than other untargeted cellular antioxidants. Coenzyme Q10 (CoQ10; ubiquinone) is a component of the mitochondrial respiratory chain with well-described antioxidant properties. The renoprotective roles of CoQ10 have been demonstrated in type 2 diabetes through preservation of mitochondrial function, such as normalizing ATP production, attenuating renal mitochondrial hydrogen peroxide production, and decreasing mitochondrial membrane potential [118]. CoQ10 is currently under phase III clinical trials for mitochondrial disorders (NCT00432744) and Parkinson’s disease (NCT00740714). mitoTEMPO, a triphenylalkylphosphonium cation (TPP+)-conjugated mitochondria-targeted antioxidant, which improves mitochondrial function by inhibiting mtROS, restoring renal mtDNA level and mitochondrial mass, and increasing the generation of ATP, can alleviate key features of IRI-induced AKI and diabetic nephropathy [94,145,146,147]. Treatment with mitoTEMPO also recovered other indicators of mitochondrial function such as PGC-1α and ATP5a-1 levels and the mitochondrial length/width ratio in ischemic reperfusion induced AKI [145]. Mitochondrial coenzyme Q (MitoQ), another compound of piperidine nitroxide conjugated to a TPP+ and a derivative of CoQ10, has been reported to attenuate renal dysfunction in several types of AKI and CKD by sequestering ROS. In IRI-AKI, administering MitoQ to mice intravenously 15 min prior to ischemia protected the kidney from damage and reduced the severity of IR injury to the kidney by decreasing oxidative damage [148]. In CKD model, MitoQ treatment decreased urinary albumin, interstitial fibrosis, and glomerular damage by decreasing mitochondrial ROS in the Ins2(+/)⁻(AkitaJ) mouse model (Akita mice) of Type 1 diabetes [119]. Moreover, MitoQ was shown to be safe in clinical trials with Parkinson’s disease (NCT00329056), fatty acid disease (NCT01167088), and hepatitis C (NCT00433108), and is currently under clinical trial (NCT02364648) for CKD. In addition to TPP+-conjugated drugs, other Mitochondria-targeted antioxidants, such as the SkQ group including plastoquinonyl-decyl-triphenylphosphonium (SkQ1) and 10-(6’-plastoquinonyl) decylrhodamine 19 (SkQR1), also present a protective effect in kidney injury [120,121]. Curcumin is thought to act as an antioxidant, which could affect various pathways relevant to the development of CKD [122,123]. In CKD model including five-sixth nephrectomy (5/6NX) and db/db mice, treatment with curcumin improved renal function through alternating mitochondria biogenesis and mitochondrial dynamics [124,149]. Curcumin is under phase III clinical trials for the treatment of Leber hereditary optic neuropathy (NCT00528151). Taken together, these studies suggest that mitochondrially targeted antioxidants represent a novel approach to prevent or attenuate kidney injury.

## 7. Conclusions and Future Directions

Mitochondrial dysfunction in renal cells plays a critical role in the pathophysiology of AKI and CKD as well as AKI to CKD transition. Various aspects of mitochondrial biology, including mitochondrial structure, dynamics, and biogenesis are involved in the progression of AKI and CKD. In addition, mitochondrial oxidative stress, and its crosstalk with other organelles, such as ER, also contribute to AKI, CKD, and AKI to CKD transition. Recent understanding of mitochondrial biology and function under the conditions of kidney diseases have revealed the mechanisms and prospective role of mitochondria-based drugs, named as mitochondria-targeting therapeutic agents, for the treatment of these kidney diseases, which may have translational potential in the future.

We should point out that even though the current evidence is encouraging, the understanding of various aspects of mitochondrial biology and function is still quite immature. For example, it remains unclear how mtDNA abnormalities are relevant to kidney injury in AKI and CKD. In addition, the clinical manifestations of mitochondrial dysfunction in kidney diseases vary in terms of symptoms, severity, and age of onset. Moreover, though studies have shown that mitochondrial dysfunction contributes to AKI and CKD, and preclinical studies suggest that mitochondria targeting therapies have the potential for prevention and management of these renal diseases, there are no translational studies showing the clinical relevance and applications of these mechanisms in humans. Thus, it is necessary to better understand mitochondrial biology and function with a focus on the mechanisms of upstream regulators and downstream effectors of mitochondrial dysfunction in renal cells and kidney diseases. We expect that these findings may translate into future therapeutic options to ameliorate renal injury and delay the progression of AKI and CKD.

## Figures and Tables

**Table 1:** The therapeutic compounds that target mitochondrial dysfunction.

Therapeutics	Mechanism(s) of Action	Experimental Model	Clinical Trial	References
Cardiolipin protection				
SS-31	Binds to cardiolipin, prevents peroxidase activity and improves mitochondrial respiration and ATP production; Inhibits cytochrome c release; Normalizes mitochondrial potential (ΔΨm)	IRI-AKI, UUO, and DKD	Mitochondrial myopathy (NCT02367014) Age-related skeletal muscle mitochondrial dysfunction (NCT02245620)	[80,96,97,98]
SS-20	Reduces mitochondrial matrix swelling and preserves cristae membranes; Increases ATP and reduces ROS	IRI-AKI		[99]
Fission inhibitor				
Mdivi-1	Selectively inhibits Drp1; Induces mitochondria fusion and increases ATP production	IRI-AKI, ADPKD, and UUO		[100,101,102]
Biogenesis activator				
SRT1720	Sirt-1 activator; Restores renal expression of PGC-1α, mitochondrial mass, ATP levels	IRI-AKI and UUO		[103,104]
Resveratrol	Sirt-1 activator; Restores mitochondrial respiratory capacity and decreases the production of mtROS and lipid peroxidation	Hemorrhagic shock induces AKI and db/db mice		[20,71,105,106,107]
AICAR	AMPK activator; Attenuates nitrosative stress and monocyte/macrophage infiltration	IRI-AKI and UUO		[13,108,109]
Formoterol	Agonist of β2-adrenoreceptor, increase PGC-1α synthesis and induces mitochondrial biogenesis by increasing mtDNA copy numbers, oxygen consumption rate	IRI-AKI and db/db mice		[110,111,112]
Fenofibrate/Bezafibrate	Peroxisomal proliferator-activated receptor (PPAR)-α agonist, lipid lowering, increases mitochondrial biogenesis	CKD and DKD	Diabetic complications (CVD and DKD) (FIELD, ISRCTN 64783481)	[113]
mPTP inhibitor				
CSA	Interacts with cyclophilin D; Suppresses mPTP opening and mitochondria swelling	IRI-AKI	Acute myocardial infarction (NCT01595958) Severe traumatic brain injury (NCT01825044)	[114]
TDZD-8	Selectively inhibits GSK-3β; Diminishes MPT	Drug-induced AKI and IRI-AKI		[115,116,117]
Antioxidants				
CoQ10	Normalizes ATP production, attenuates mtROS and decreases mitochondrial ΔΨm	db/db mice	Mitochondrial disorders (NCT00432744) Parkinson disease (NCT00740714)	[118]
MitoQ	ROS scavenger; Antioxidant concentrates at matrix in a ΔΨm-dependent manner	IRI-AKI Ins2(+/)⁻(AkitaJ) mice	Parkinson’s disease (NCT00329056) Fatty acid disease (NCT01167088) Hepatitis C (NCT00433108) CKD (NCT02364648)	[66,119]
SkQR1	Antioxidant and decreased mitochondrial ΔΨm	IRI-AKI and gentamycin-induced renal failure		[120,121].
Curcumin	Antioxidant; Alteration of mitochondrial dynamics and bioenergetics.	5/6NX mice and db/db mice	Leber hereditary optic neuropathy (NCT00528151)	[122,123,124]

ATP: adenosine triphosphate; ΔΨm: mitochondrial membrane potential; IRI-AKI: ischemic reperfusion induced acute kidney injury; UUO: unilateral ureter obstruction; DKD: diabetic kidney diseases; ROS: reactive oxygen species; Mdivi-1: Mitochondrial division inhibitor-1; ADPKD: autosomal dominant polycystic kidney disease; Drp1: dynamin related protein 1; Sirt1: Sirtuin 1; PGC-1α: PPARgamma-coactivator-1α; AMPK: AMP-activated protein kinase; mtROS: mitochondria reactive oxidative species; mtDNA: mitochondria DNA; CKD: chronic kidney disease; mPTP: mitochondrial permeability transition pore; CSA: Cyclosporine-A; TDZD-8: 4-benzyl-2-methyl-1,2,4-thiadiazolidine-3,5-dione; GSK-3β:, glycogen synthase kinase 3β; CoQ10: Coenzyme Q10 MitoQ: mitochondrial coenzyme Q; SkQR1: 10-(6′-plastoquinonyl) decylrhodamine 19; 5/6NX: five-sixth nephrectomy.
